# Membrane potential independent transport of NH_3_ in the absence of ammonium permeases in *Saccharomyces cerevisiae*

**DOI:** 10.1186/s12918-016-0381-1

**Published:** 2017-04-17

**Authors:** Hugo F. Cueto-Rojas, Nicholas Milne, Ward van Helmond, Mervin M. Pieterse, Antonius J. A. van Maris, Jean-Marc Daran, S. Aljoscha Wahl

**Affiliations:** 1 Department of Biotechnology, Delft University of Technology, van der Maasweg 9, 2629HZ Delft, The Netherlands; 2 Present Address: Evolva Biotech A/S, Lersø Parkallé 42, 2100 København Ø, Denmark; 3 Present Address: Nederlands Forensisch Instituut (NFI), Laan van Ypenburg 6, 2497 GB Den Haag, The Netherlands; 4 Division of Industrial Biotechnology, School of Biotechnology, KTH Royal Institute of Technology, AlbaNova University Center, SE 106 91 Stockholm, Sweden

**Keywords:** Intracellular ammonium, Metabolomics, Ammonium transport, Central nitrogen metabolism, Ammonia passive diffusion, Thermodynamics

## Abstract

**Background:**

Microbial production of nitrogen containing compounds requires a high uptake flux and assimilation of the N-source (commonly ammonium), which is generally coupled with ATP consumption and negatively influences the product yield. In the industrial workhorse *Saccharomyces cerevisiae*, ammonium (NH_4_^+^) uptake is facilitated by ammonium permeases (Mep1, Mep2 and Mep3), which transport the NH_4_^+^ ion, resulting in ATP expenditure to maintain the intracellular charge balance and pH by proton export using the plasma membrane-bound H^+^-ATPase.

**Results:**

To decrease the ATP costs for nitrogen assimilation, the Mep genes were removed, resulting in a strain unable to uptake the NH_4_^+^ ion. Subsequent analysis revealed that growth of this ∆*mep* strain was dependent on the extracellular NH_3_ concentrations. Metabolomic analysis revealed a significantly higher intracellular NH_X_ concentration (3.3-fold) in the ∆*mep* strain than in the reference strain. Further proteomic analysis revealed significant up-regulation of vacuolar proteases and genes involved in various stress responses.

**Conclusions:**

Our results suggest that the uncharged species, NH_3_, is able to diffuse into the cell. The measured intracellular/extracellular NH_X_ ratios under aerobic nitrogen-limiting conditions were consistent with this hypothesis when NH_x_ compartmentalization was considered. On the other hand, proteomic analysis indicated a more pronounced N-starvation stress response in the ∆*mep* strain than in the reference strain, which suggests that the lower biomass yield of the ∆*mep* strain was related to higher turnover rates of biomass components.

**Electronic supplementary material:**

The online version of this article (doi:10.1186/s12918-016-0381-1) contains supplementary material, which is available to authorized users.

## Background

A significant number of fuels and commodity chemicals have the potential to be produced in bio-refineries using microbial fermentation, which represents a more sustainable alternative to current oil-based production [1]. The increasing interest in microbial-based production is best exemplified by the intensive research efforts to improve the productivity and yield of a vast range of different compounds produced by *Saccharomyces cerevisiae* [2, 3] and other industrial workhorses. Nevertheless, while the number of compounds produced at industrial scale by *S. cerevisiae* is increasing, the production of nitrogen-containing compounds using *S. cerevisiae* is significantly under-represented, with heterologous protein production being the only known example [3].

Nitrogen-containing compounds represent an economically relevant class of commodity chemicals that includes amino acids such as L-lysine and L-glutamate, diamines such as 1,5-diaminopentane (cadaverine) and 1,4-diaminobutane (putrescine), and relevant synthesis precursors such as caprolactam. Their microbial production is currently performed under aerobic conditions using bacteria, most commonly *Corynebacterium glutamicum* and *Escherichia coli* [4–6].

Along with bacteria, *S. cerevisiae* is seen as an attractive host organism for industrial fermentation due to its fast anaerobic conversion of sugar to product, its resistance to phage attack, and its robustness under common industrial conditions [7]. When using *S. cerevisiae* for the production of nitrogen-containing compounds, the process should preferably occur under anaerobic conditions [8] if this is permitted by the thermodynamics and biochemistry of the product pathway. Anaerobic conditions are favorable not only in terms of the resulting fermentation costs, but also in terms of the product yield [9]. Under such conditions, however, the energy supply relies solely on substrate-level phosphorylation, limiting the amount of ATP available for growth and maintenance. Consequently, the anaerobic production of nitrogen-containing compounds should result in net ATP formation and it is essential that the N-source be transported and assimilated using ATP-independent mechanisms.

Urea and ammonium are the most common N-sources used industrially in *S. cerevisiae* fermentations. Previously, we presented a novel strategy for achieving ATP-independent urea assimilation in *S. cerevisiae* [10]. While urea is an attractive nitrogen source, ammonium is more commonly used in industrial fermentation and is also present in plant hydrolysates used for second-generation chemical production [11, 12]. Mechanisms for ATP-neutral ammonium transport and assimilation would have significant relevance for the anaerobic production of nitrogen-containing compounds. Ammonia (NH_3_) protonates in aqueous solutions to produce the ammonium ion (NH_4_^+^), the sum of these two species, NH_3_ and NH_4_^+^, will be described henceforth as NH_X_. With a *pKa* of 9.25, under biologically relevant conditions (between pH 3 and 7), the ratio NH_3_/NH_4_^+^ equals 10^pH-9.25^, meaning that the vast majority of the NH_X_ is present as the charged ammonium species (NH_4_^+^).

In *S. cerevisiae*, NH_4_^+^ is taken up by the ammonium permeases Mep1, Mep2, and Mep3, which belong to the Amt class of proteins that use the negative membrane potential as their thermodynamic driving force [13]. The evolutionary advantage of this transport mechanism, compared with passive diffusion, is a higher transport rate. And, due to the negative cytosolic membrane potential, accumulation of intracellular NH_X_ is favored. However, one H^+^ must be exported from the cytosol by the plasma-membrane-bound H^+^-ATPase Pma1 [14] to recover the proton motive force (pmf) and charge homeostasis after NH_4_^+^ import, and subsequent assimilation of NH_3_ [8]. The deletion of the ammonium permease genes Mep1, Mep2, and Mep3 results in a viable strain able to grow on ammonium concentrations above 5 mM. Previously, it has been assumed that there are additional ammonium transporters [15] or that there is non-specific transport through potassium channels [16]. However, we here present an alternative hypothesis: that the uncharged NH_3_ species can diffuse into the cell. If this were correct, it would result in ATP-independent NH_X_ uptake and consequently reduce the demand for ATP demand. Previous experimental observations in synthetic bilayer lipid membranes suggest that the NH_3_ apparent permeability coefficient is *P*_*1a*_ = 1.728 m/h (48 × 10^−3^ cm/s) [17], which indicates that cell membranes are indeed permeable to NH_3_.

Here, we study the NH_X_-uptake mechanism in a ∆*mep S. cerevisiae* strain, and assess the impact of the deletion of Mep1, Mep2, and Mep3 on the physiology of *S. cerevisiae*. Proteomic and metabolomic measurements are used to investigate the global impact of the changed NH_X_-uptake mechanism on cellular physiology.

## Methods

### Strains and maintenance

All *Saccharomyces cerevisiae* strains used in this study (Table 1) were derived from the CEN.PK strain family background [18, 19], details about strain contraction are found in Additional file 1. Frozen stocks of *E. coli* and *S. cerevisiae* were prepared by addition of glycerol (30% (v/v)) to exponentially growing cells followed by aseptic storage of 1 mL aliquots at -80 °C. Cultures were grown at 30 °C either in synthetic medium [20] with 20 g/L glucose as carbon source and appropriate growth factors [21], or complex medium containing 20 g/L glucose, 10 g/L Bacto yeast extract and 20 g/L Bacto peptone. If required for anaerobic growth Tween-80 (420 mg/L) and ergosterol (10 mg/L) were added. Agar plates were prepared as described above but with the addition of 20 g/L agar (Becton Dickinson B.V. Breda, The Netherlands).

**Table 1:** Strains used in this study

Name	Relevent genotype	Origin
CEN.PK113-3B	*MATalpha ura3-52 his3-D1 LEU2 TRP1 MAL2-8c SUC2*	[18]
CEN.PK113-3B-Δmep1	*MATalpha ura3-52 his3-D1 LEU2 TRP1 MAL2-8c SUC2 mep1::loxP-*KanMX4-loxP	This study
CEN.PK113-3B-Δmep1, Δmep 2	*MATalpha ura3-52 his3-D1 LEU2 TRP1 MAL2-8c SUC2 mep1::loxP-*KanMX4-*loxP mep2::loxP*-NatNT2-*loxP*	This study
CEN.PK113-3B-Δmep1, Δmep 2, Δmep 3	MATalpha ura3-52 his3-D1 LEU2 TRP1 MAL2-8c SUC2 *mep1::loxP*-KanMX4-*loxP mep2::loxP*-NatNT2-loxP *mep3::loxP-Ura3-loxP*	This study
CEN.PK113-3B-Δmep1, Δmep 2, Δmep 3-Cure	*MATalpha ura3-52 his3-D1 LEU2 TRP1 MAL2-8c SUC2 mep1::loxP*-*loxP mep2::loxP-loxP mep3::loxP-loxP*	This study
IMZ351	*MATalpha ura3-52 his3-D1 LEU2 TRP1 MAL2-8c SUC2 mep1::loxP-loxP mep2::loxP-loxP mep3::loxP-loxP* pUDE199 (*HIS3 URA3*)	This study
IME169	*MATalpha ura3-52 his3-Δ1 LEU2 TRP1 MAL2-8c SUC2* pUDE199 (*HIS3 URA3*)	This study

### Strain cultivation

#### Shake flask cultivation

*S. cerevisiae* strains were grown in synthetic medium [22]. Cultures were grown in either 500 mL or 250 mL shake flasks containing 100 mL or 50 mL of medium, respectively, and incubated at 30 °C in an Innova incubator shaker (New Brunswick Scientific, Edison, NJ) at 200 rpm.

#### Aerobic nitrogen-limited chemostat cultivation

Controlled aerobic, nitrogen limited chemostat cultivations were carried out at 30 °C in 7 L bioreactors (Applikon Biotechnology B.V., Delft, the Netherlands) with a working volume of 4 L. Chemostat cultivations were preceded by a batch phase using the same synthetic medium as used for the feed. Continuous cultivation was initiated at a dilution rate of 0.05 h^−1^; synthetic nitrogen-limited medium was used modified from [23], which contained: 130 g/L glucose, 25 g/L ethanol, 3.48 g/L NH_4_H_2_PO_4_, 1.14 g/L MgSO_4_ · 7H_2_O, 6.9 g/L KH_2_PO_4_, 0.3 g/L Antifoam C, with the appropriate growth factors added accordingly [21] (vitamin solution 2 mL/L and trace element solution 2 mL/L), ethanol was added to the medium to avoid potential oscillations. The medium was designed to sustain a biomass concentration of up to 8 g/L in nitrogen-limited anaerobic conditions for the wild type (CEN.PK113-7D) strain. The temperature and stirring speed were kept constant at 30 °C and 500 rpm, respectively. The reactor had an overpressure of 0.3 bar, and an aeration rate of 0.5 vvm was used to keep the dissolved oxygen level above 80%. Dissolved oxygen tension (DOT) was monitored online using an oxygen probe (Mettler-Toledo, Tiel, The Netherlands), and a combined paramagnetic/infrared analyser (NGA 2000, Fisher-Rosemount, Hasselroth, Germany) was used to measure CO_2_ and O_2_ fractions in the off-gas. During the batch phase and the first steady state the pH was kept constant at a value of 5 with automatic additions of 4 M KOH or 2 M H_2_SO_4_; after reaching steady state and sampling, the pH control was changed to maintain a constant value of 6, while keeping the dilution rate constant; the same operation was performed for the switch from pH = 6 to pH = 7. All samples were taken at steady state between three and seven volume changes after switching on the medium addition or pH changes.

### Sampling and sample preparation

#### Extracellular sampling

For aerobic nitrogen limited chemostats, samples of approx. 2 mL were quenched using cold steel beads [24], and filtered using 0.45 μm disc filters (Milipore). Samples for residual ammonium determination were prepared by mixing 80 μL of sample with 20 μL of internal standard (500 μmol/L ^15^N-NH_4_Cl) and quantified according to [25]. All samples were stored at -80 °C until further analysis.

#### Intracellular sampling

Samples containing approximately 1.2 g broth were obtained using a dedicated setup, as described by [26], quenched in 6 mL of -40 °C methanol 100%, and after weighing to accurately determining the mass of each sample, these were centrifuged for 5 min at 10,000 g and -19 °C. The pellet was recovered and resuspended in 6 mL -40 °C methanol 100%; then centrifuged again for 5 min at 10,000 g and -19 °C [27].

#### Intracellular ammonium extraction

The biomass pellet obtained from *Intracellular sampling* was recovered, 3.5 mL of Methanol-acetate buffer 10 mM (pH = 5) 50%(v/v) pre-chilled at -40 °C was added, and then 120 μL of U-^13^C- cell extract with labeled urea (intracellular metabolites samples) or 120 μL of ^15^N- NH_4_Cl 500 μmol/L (intracellular ammonium samples) were added as internal standard. Afterwards, 3.5 mL of Chloroform 100% pre-chilled at -40 °C was added in order to extract intracellular metabolites according to [25]. Samples for quantification of intracellular ammonium were extracted using exclusively this method.

#### Intracellular metabolite extraction

The biomass pellet (*Intracellular sampling*) was recovered by addition of 3.5 mL Methanol-MilliQ water 50% (v/v) pre-chilled at -40 °C and 120 μL of U-^13^C- cell extract. 3.5 mL of chloroform 100% pre-chilled at -40 °C was added in order to extract intracellular metabolites as described by [27].

### Analytical methods

#### Micro-titer plate assays

Ninety-six well plate assays were prepared by adding 100 μL of synthetic medium with 20 g/L glucose, Tween-80 (420 mg/L) and ergosterol (10 mg/L). The initial pH of the medium was adjusted using 2 M HCl and 2 M KOH. (NH_4_)_2_SO_4_ was used as the nitrogen source and the SO_4_^2−^ concentration was kept constant at 38 mM by addition of K_2_SO_4_ to compensate for the decrease in SO_4_^2−^ from (NH_4_)_2_SO_4_. Cells were inoculated in each well to a starting OD_660_ of 0.1. Plates were covered with Nunc™ sealing tape (Thermo Scientific) and incubated at 30 °C with constant shaking at 200 rpm. OD660 was measured regularly in a GENios pro plate reader (Tecan Benelux, Giessen, The Netherlands).

#### Metabolite quantification

Quantification of intracellular trehalose, glycolytic, TCA cycle and PPP intermediates was performed as described by [28]; amino acids were quantified according to [29], nucleotides as described in [30] and coenzymes were measured using LC-MS/MS as reported by [31]. Intra- and extracellular ammonium was quantified using ultra-high performance liquid chromatography with isotope dilution mass spectrometry (UHPLC-IDMS) as described by [25]. Quantification of extracellular metabolites was performed using HPLC as described in [32]. Cellular concentrations were estimated using the metabolite content per g_CDW_ (μmol/g_CDW_) and the average cell volume including dry matter (mL_WC_/g_CDW_), which was determined using a Z2 Coulter counter (50 μm aperture, Beckman, Fullerton, CA) [33].

#### Proteomic analysis

U-^13^C-labelled *S. cerevisiae* biomass was prepared as described by [34] and used as internal standard for relative protein quantification. Cell suspensions of the sample biomass and internal standard were mixed 1:1 based on the OD_600_, washed with milli-Q and freeze-dried. Proteins were extracted by grinding the freeze-dried biomass with pestle and mortar, which were precooled with liquid nitrogen. After grinding, 2 mL of 50 mM phosphate buffer (PBS) with 200 mM NaOH was added to extract proteins. The soluble protein fraction was separated from the cell debris by centrifugation at 13,300 rpm for 15 min. Proteins were precipitated overnight in cold acetone at -20 °C by adding 4 parts of cold acetone to 1 part of protein solution. After washing and drying the protein pellet was dissolved in 400 μL of 100 mM ammonium bicarbonate (ABC) with 6 M urea. Of this solution, 20 μL was further processed; proteins were reduced by addition of tris(2-carboxyethil)phosphine (TCEP) to a final concentration of 10 mM and incubating for 60 min at room temperature. Proteins were alkylated by addition of Iodoacetamide (IAM) to a final concentration of 10 mM and incubating for 60 min at room temperature. Prior to digestion the protein solution was 6 times diluted by addition of 100 μL of 100 mM ABC to dilute the urea concentration to 1 M. Proteins were digested by addition of trypsin (trypsin singles, proteomics grade, Sigma-Aldrich) in a 1:100 ratio and incubating at 37 °C for 16 h. The digested protein mixture was purified and concentrated using an in-house made SPE pipette tip using 5 μm particles of Reprosil-Pur C_18_-Aq reversed phase material (Dr. Maisch GmbH, Ammerbuch-Entringen, Germany).

Digested peptides were separated using nanoflow chromatography performed using a vented column system essentially as described by [35] and a 2-dimensional precolumn (RP-SCX-RP). Analytical columns of 50 μm id were prepared with a 1 mm Kasil frit and packed with 5 μm particles of Reprosil-Pur C_18_-Aq reversed phase material to a length of 40 cm. The capillary RP-SCX-RP precolumn of 150 μm id was prepared with a 1 mm Kasil frit and packed with 5 μm particles of Reprosil-Pur C18-Aq reversed phase material to a length of 17 mm, 5 μm particles of PolySulfoethyl a strong cation exchange material for 60 mm and again 5 μm particles of Reprosil-Pur C_18_-Aq reversed phase material for 17 mm (total length 94 mm). The different column materials were kept separated from each other by insertion of a piece of glass wool. The used LC equipment and solvents were similar to [36]. Each sample analysis consisted of six fractionations. In the first fraction the peptides are injected and trapped on the precolumn by applying 100% solvent A for 10 min. Then a first linear gradient was applied from 4 to 35% B in 75 min. After this, a linear gradient to 80% B was followed for 6 min and then 3 min of 80% B. Finally the column was reconditioned for 26 min with 100% A. In the following 5 fractionations, peptides were eluted by 10 μL injections of respectively 5, 10, 50, 250 or 1000 mM ammonium formate pH 2.6 from the autosampler (followed by 100% A for 10 min). Again a first linear gradient was applied from 4 to 35% B in 75 min, followed by a second linear gradient to 80% B for 6 min and then 3 min of 80% B. After each fraction the column was reconditioned for 26 min with 100% A. This results in six fractionations per sample with a total run-time of 12 h per sample. For each analysis ~10 μg of protein was injected.

Mass spectrometry was performed using a protocol derived from [36] and similar to [37]. Briefly explained, full scan MS spectra (from m/z 400–1500, charge states 2 and higher) were acquired at a resolution of 30,000 at m/z 400 after accumulation to a target value of 10^6^ ions (automatic gain control). Nine data-dependent MS/MS scans (HCD spectra, resolution 7,500 at m/z 400) were acquired using the 9 most intense ions with a charge state of 2+ or higher and an ion count of 10,000 or higher. The maximum injection time was set to 500 ms for the MS scans and 200 ms for the MS/MS scan (accumulation for MS/MS was set to target value of 5 × 10^4^). Dynamic exclusion was applied using a maximum exclusion list of 50, one repeat count, repeat duration of 10 s and exclusion duration of 45 s. The exclusion window was set from −10 to + 10 ppm relative to the selected precursor mass.

Data processing and analysis was performed similarly to [36]. Briefly, MS/MS spectra were converted to Mascot Generic Files (MGF) using Proteome Discoverer 1.4 (ThermoFisher Scientific) and DTASuperCharge version 2.0b1 [38]. MGF’s from the 6 SCX fractions of the same sample were combined using MGFcombiner version 1.10 [38]. The samples were analyzed with Mascot v2.2.02 search engine (Matrix Science, Boston, MA, USA). As reference proteome the Uniprot [39] proteome of *Saccharomyces cerevisiae* strain ATCC 204508/288c (ID: UP000002311; 6634 sequences) was used.

Carbamidomethyl cysteine was set as a fixed modification and oxidized methionine as a variable modification. Trypsin was specified as the proteolytic enzyme, and up to three missed cleavages were accepted. Mass tolerance for fragment ions was set at 0.05 Da and for precursor peptide ions at 10 ppm. Peptides with Mascot score <10 were removed and only the highest scoring peptide matches for each query listed under the highest scoring protein (bold red) were selected. Proteins were quantified using MSQuant version 2.0b7 [38] by importing the Mascot results html file with the corresponding raw mass spectrometric data files. MSQuant automatically calculated peptide and protein ratios by using a ^13^C quantitation method (in quantitationmodes.xml), containing 7 modifications based on the amount of carbon atoms each amino acid contains. The difference in mass between ^12^C and ^13^C is 1.00335 Da. Resulting in mass shifts of 2 (glycine), 3 (ASC), 4 (NDT), 5 (EQMPV), 6 (RHILK), 9 (FY) or 11 (W) carbon atoms. Quantification was restricted to peptides with Mascot score ≥25, it is considered that a protein is up regulated when the concentration of protein is at least 50% higher in one strain compared to the other, growing at the same environmental condition. On the other hand, proteins identified with 2 or more confidence peptides with Mascot score ≥25 in one strain but not in the other are considered “unique proteins”.

## Results

### Effect of extracellular NH_3_ concentration on growth rate

**Fig. 1:**
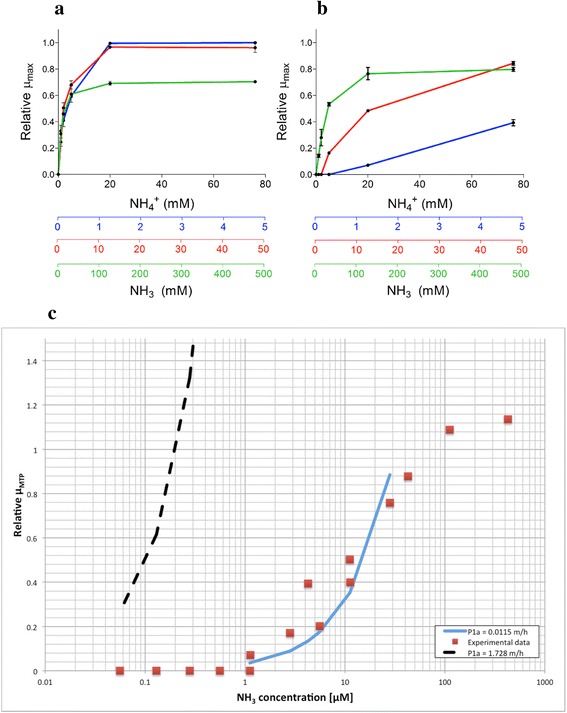
Relative specific growth rate in micro-titer plate (μ_MTP_) of (**a**) IME169 (reference strain) and (**b**) IMZ351 (*Δmep*) at different pH and extracellular NH_X_-concentrations, pH = 5 (*blue)*, pH = 6 (*red*) and pH = 7 (*green*) in synthetic medium with glucose supplemented with Tween-80 (420 mg/L) and ergosterol (10 mg/L). **c** Relative specific growth rates in micro-titer plate (μ_MTP_) of IMZ351 at different NH_3_-concentrations irrespective of extracellular pH. Growth rates were determined from exponentially growing cells cultured in 100 μL synthetic medium in 96 well plates with OD_660_ measurements taken every 15 min. The SO_4_ concentration was kept constant at 38 mM by supplementation with K_2_SO_4_. Data are presented as averages and standard deviations of duplicate experiments, relative to the average growth rate of IME169 at pH = 5, with 76 mM NH_4_^+^ (μ_max_ = 0.21 h^−1^). The continuous blue line represents an apparent permeability coefficient of 0.0115 m/h (0.32 10^−3^ cm/s), calculated using least squares in the linear region of the experimental data (R^2^ = 0.73); the discontinuous black line shows the trend of the growth rate if an apparent permeability coefficient of 1.728 m/h (48 10^−3^ cm/s) is assumed [17]

To identify whether the elimination of the ammonium permeases will eliminate NH_4_^+^ uptake and result in NH_3_ diffusion as the sole mechanism, all permeases (Mep1, Mep2, and Mep3) were knocked-out. This resulted in strain IMZ351 (Additional file 1). Relative specific aerobic growth rates in micro-titer plate (μ_MTP_) of IMZ351 (*Δmep*) and the control strain IME169 (Mep1, Mep2, Mep3) were compared at varying initial pH values and (NH_4_)_2_SO_4_ concentrations under aerobic conditions (Fig. 1). The concentration of NH_3_ at a given (NH_4_)_2_SO_4_ concentration is dependent on the extracellular pH. Note that increasing the pH significantly increases the NH_3_ concentration. However, because the *pKa* = 9.25 strongly favors the charged form, the NH_4_^+^ concentration remains relatively unchanged, at between pH 3 and 7.

The growth rate of IME169 reached a maximum at approximately 20 mM NH_X_. The growth rate was negatively affected by increasing pH values (Fig. 1a), an expected effect caused by the deviation from the optimum pH =5. On the other hand, it was observed that the strain IMZ351 increased its growth rate with increasing pH values (Fig. 1b). Consequently, plotting the specific growth rate as a function of the NH_3_ concentration revealed a clear correlation between the two variables (Fig. 1c), indicating that the growth of IMZ351 was dependent on NH_3_ concentration whereas IME169 growth was dependent on NH_4_^+^ concentrations. This supports the hypothesis that deletion of Mep proteins leads to a change in the main uptake mechanism, from NH_4_^+^ uniport to NH_3_ diffusion. Clearly, diffusion is also present in the wild type. But, because of the electrochemical-based driving force, the concentration gradient for diffusion is actually in the direction of export rather than import. Thus, NH_X_ uptake can only take place in the Mep-dependent transport mechanism.

If the Δ*mep* strain (IMZ351) indeed relied on diffusion of NH_3_ to supply nitrogen to the cell, then the specific rate of N-uptake (*-q*_*N*_, in mol N/g_CDW_/h) is dependent on the NH_3_ concentration gradient between extracellular space and cytosol ([*NH*_*3*_]_*EC*_ *-*[*NH*_*3*_]_*cyt*_, in mmol/L). The rate of NH_3_ uptake can be described by the apparent permeability coefficient (*P*_*1a*_, in m/h) of the membrane, the biomass specific mass transfer area (*a*_*m*_, in this study 3.22 m^2^/g_CDW_), and the concentration gradient: − *q*_*N*_ = *P*_1*a*_ *a*_*m*_([*NH*_3_]_*EC*_ − [*NH*_3_]_*cyt*_). Under nitrogen-limited conditions, the growth rate will be dependent on the extracellular NH_3_ concentration, i.e., *µ* = *χ*_*N*_^− 1^ − *q*_*N*_, with *χ*_*N*_ representing the biomass N-content (usually 0.148 mol N/C-mol biomass or 5.60 × 10^−3^ mol N/g_CDW_ [40]).

Based on this dependency, the NH_3_ permeability coefficient for batch conditions can be estimated. Assuming that the NH_3_ extracellular concentration is much higher than the cytosolic concentration, the previous dependency can be approximated by − *q*_*N*_ = *P*_1*a*_ *a*_*m*_[*NH*_3_]_*EC*_. With the array of measured μ_MTP_ as a function of the initial NH_3_ concentration (Fig. 1c), the NH_3_ permeability coefficient is estimated as *P*_*1a*_ = 0.01 m/h. This value is two orders of magnitude below values reported in literature. This large deviation from the permeability measured in vitro could be due to different membrane compositions, but above all it is the assumption of negligible intracellular NH_X_ concentration, which has an impact on the value obtained. The estimated value therefore represents the lower limit of permeability rather than a precise measure.

### Intracellular and extracellular NH_X_ ratios under N-limiting conditions

The micro-titer assay described above showed a clear link between the extracellular NH_3_ concentration and the growth rate of IMZ351, but these results cannot provide insights into the intracellular metabolism. Moreover, the absence of pH control and monitoring of dissolved oxygen concentration could potentially bias these results. In order to perform a detailed analysis of the resulting strain physiology in response to different mechanisms of NH_X_ assimilation, aerobic N-limited chemostat cultures were carried out at varying pH values (pH = 5, pH = 6, pH = 7). Extracellular and intracellular metabolite measurements were performed at each steady-state condition. The aerobic N-limited conditions were selected to observe the energetic effect of NH_3_-diffusion based on differences in specific oxygen consumption rates (-*q*_*O2*_) between strains. Additionally, the use of N-limited conditions reduced the residual NH_X_ and so increased the accuracy of the intracellular NH_X_ measurements.

To ensure that the differential effect of pH and NH_X_ concentration between the two strains were indeed based on differences in transport mechanisms, the cytosolic/extracellular NH_X_ ratio was determined for both strains. If NH_4_^+^ were the only species being transported into the cell, then the uptake rate and the cytosolic/extracellular NH_X_ ratio at steady state under N-limiting conditions would depend on the membrane potential. By contrast, if NH_3_ were the only species being transported into the cell, then the NH_X_-uptake rate and cytosolic/extracellular NH_X_ ratio would depend only on the NH_3_ concentration gradient across the cell membrane (Additional file 1). In other words, the two transport mechanisms can be discriminated on the basis of their different cytosolic/extracellular NH_X_ ratios (Table 2). Furthermore, because the growth rate is similar for all cultivations and NH_X_ is the limiting substrate, the cytosolic concentration of this compound was expected to be similar (if not the same), regardless of the transport mechanism, to support the same downstream nitrogen fluxes.

**Table 2:** Intracellular and extracellular NH_X_ concentrations of IME169 (reference strain) and IMZ351 (*Δmep*) measured at steady state at varying pH values from aerobic N-limited chemostats in synthetic medium with glucose at a dilution rate of 0.05 h^−1^ and the corresponding NH_X_ IC/EC ratios. For calculation of predicted intracellular/extracellular ratios with compartmentalization three compartments were considered: cytosol, mitochondria and vacuole. The ratios were calculated as the maxima and minima of a sensitivity analysis where the following critical variables were considered: vacuolar volumes (between 25 and 14% intracellular volume), cytosolic pH (between 6 and 7) and vacuolar pH (between 4 and 5.5). The data represent average and mean deviation of triplicates

Strain	pH	Average cell volume (mL_IC_/g_CDW_)	Biomass concentration (g_CDW_/L_broth_)	Intracellular NH_X_ (mmol/L_IC_)	Extracellular NH_X_ (mmol/L_EC_)	Measured IC/EC ratio	Predicted IC/EC equilibrium ratio range	
							Maximum	Minimum
IME169 Uniport NH_4_^+^	5.0	2.59 ± 0.04	7.00 ± 0.02	1.74 ± 0.14	0.008 ± 0.001	219 ± 39	5.44 × 10^3^	108
	6.0	2.43 ± 0.04	7.45 ± 0.01	3.16 ± 0.16	0.011 0.003	302 ± 40	5.44 × 10^4^	1.09 × 10^3^
	7.0	2.62 ± 0.02	7.73 ± 0.03	3.33 ± 0.09	0.013 ± 0.001	254 ± 10	5.44 × 10^5^	1.09 × 10^4^
IMZ351 Diffusion NH_3_	5.0	2.01 ± 0.08	6.44 ± 0.01	10.5 ± 0.7	6.99 ± 0.28	1.5 ± 0.1	2.57	0.05
	6.0	2.00 ± 0.04	7.37 ± 0.04	10.9 ± 0.6	2.61 ± 0.09	4.2 ± 0.3	25.7	0.5
	7.0	2.31 ± 0.09	7.73 ± 0.01	7.48 ± 0.7	0.57 ± 0.02	13.2 ± 1.3	255	5

However, the cytosolic NH_X_ concentration cannot be measured directly. Current metabolomic approaches allow only for whole-cell quantifications, which from now on will be called intracellular (IC) measurement. In the case of NH_X_, previous works [41, 42] suggest significant accumulation and storage of NH_X_ in the vacuole, which means that the whole-cell measurement and the cytosolic concentration could differ significantly. To account for vacuolar storage, the measured NH_X_ ratios were compared with expected maximum and minimum ratios (IC/EC) based on assumptions for vacuolar diffusion (Additional file 1). Interestingly, the expected difference in ratios still allows for a clear separation of mechanisms in the presence of vacuolar storage.

In line with our hypothesis, the experimental data showed ratios with a difference of at least one order of magnitude between IME169 (Mep1, Mep2, Mep3) and IMZ351 (*∆mep*) (Table 2). For IMZ351, the IC/EC ratios measured experimentally corresponded well with the predicted ratios. However, while the IC/EC ratio for IME169 was predicted to increase with extracellular pH, it actually varied between 210 and 300 under the experimental conditions (Table 2) - which might indicate that, under these conditions, the ratio is determined by the affinity of the ammonium permeases and not by the thermodynamic driving force. Besides differences in IC/EC ratios, a substantially higher intracellular NH_X_ concentration was observed for IMZ351.

### Estimation of the NH_3_ permeability coefficient at steady state under N-limiting conditions

Under N-limiting conditions, it can be assumed that transport of the N-source is the limiting factor for growth in both strains. In IMZ351, the diffusion rate is determined, as explained earlier, by the NH_3_ permeability and the concentration gradient across the plasma membrane (([*NH*_*3*_]_*EC*_ *-*[*NH*_*3*_]_*cyt*_). While the concentration in the extracellular space ([*NH*_*3*_]_*EC*_) is measured directly, the cytosolic concentration (*[NH*_*3*_ *]*_*cyt*_) needs to be estimated from the whole-cell measurement (IC), the specific nitrogen uptake rate (-*q*_*N*_), and assumptions regarding the intracellular NH_X_ distribution (Additional file 1). Here, it is assumed that the cytosol volume represents 70% of the cell volume, the vacuolar volume is 14% and the mitochondrial volume is about 1% of the total cell volume [43].

Additionally, NH_3_ transport processes between different compartments are assumed to operate close to thermodynamic equilibrium -and since no transport proteins that could translocate NH_X_ between compartments are described in literature, passive diffusion of NH_3_ between vacuole and cytosol, as well as between cytosol and mitochondria, are assumed.

With these assumptions and measurements, a linear equation system is set up to calculate the missing variables (Additional file 1). The apparent permeability coefficient varies between 0.03 m/h and 2.73 m/h (Table 3), decreasing with pH, as has also been observed for other biological systems [44]. It should also be mentioned that, for an extracellular pH of 5, the assumptions for vacuolar size and pH have to be adjusted to 25% of the cell volume and 4.2, respectively, in order to obtain a positive NH_3_ concentration gradient between extracellular space and cytosol.

**Table 3:** Estimation of the apparent permeability coefficient of ammonium for IMZ351 (*Δmep*) into the plasma membrane

Strain	pH_EC_	pH_vac_	Cytosolic NH_3_ (μmol/L_Cyt_)	Extracellular NH_3_ (μmol/L_EC_)	Estimated Cyt/EC ratio	Apparent permeability coefficient (m/h)
IMZ351	5.0^a^	4.2^a^	0.37	0.39	0.030	2.73^a^
	6.0	4.5	1.31	1.47	0.283	0.37
	7.0	4.5	0.90	3.16	0.902	0.03

^a^ In this particular case, a numerical solution to the system of algebraic equations that estimates *P*_*1a*_ (Additional file 1) is achieved only if the vacuolar pH was 4.2 and the vacuolar volume considered was 25% of the total cell volume

### Impact of NH_3_-diffusion on the physiology and metabolic fluxes of *S. cerevisiae* under aerobic N-limiting conditions

#### Effect of diffusion on the specific consumption and production rates

The effect of NH_3_-dependent mechanism of nitrogen uptake on ATP consumption was determined based on a simple metabolic model. All relevant q-rates and physiological parameters are shown in Table 4. The ATP production rate was calculated based on the oxygen consumption rate (1.9 mol ATP/mol O_2_) and the rate of alcoholic fermentation (1 mol ATP/mol ethanol) under respirofermentative conditions, which was observed under N-limiting conditions [45]. Contrary to the expectation of a reduced ATP cost per assimilated N-mole, IMZ351 consumed more ATP per mole of N-assimilated than IME169. So secondary effects like increased N-starvation stress could lead to higher ATP consumption. This hypothesis is further supported by an observed decrease in N-conent and higher C/N consumption, together with a higher production of reserve carbohydrates (i.e., trehalose and glycogen), which are related to stress response.

**Table 4:** Overview of measured extracellular fluxes and N-content of IME169 (reference strain) and IMZ351 (*Δmep*) during N-limited aerobic chemostats in synthetic medium with glucose at a dilution rate of 0.05 h^−1^ at different extracellular pH

Strain	pH_EC_	μ	-q_S_	-q_O2_	q_CO2_	q_Ethanol_	-q_N_	N-content	Y_XS_	C/N consumption	q_ATP_	q_ATP_/-q_N_
		1/h	mmol/g_CDW_/h	mmol/g_CDW_/h	mmol/g_CDW_/h	mmol/g_CDW_/h	mmol/g_CDW_/h	mmol N/g_CDW_	g_CDW_/g_Glc_	C-mol/N-mol	mmol/g_CDW_/h	mol ATP/mol N
IME169	5.0	0.053 ± 0.001	3.862 ± 0.050	1.643 ± 0.006	7.028 ± 0.015	4.601 ± 0.223	0.251 ± 0.001	4.70 ± 0.01	0.077 ± 0.001	92.3 ± 1.3	7.72 ± 0.22	30.8 ± 0.9
	6.0	0.052 ± 0.001	3.398 ± 0.013	1.468 ± 0.004	6.157 ± 0.010	4.438 ± 0.055	0.223 ± 0.004	4.30 ± 0.08	0.085 ± 0.001	91.4 ± 1.7	7.23 ± 0.05	32.4 ± 0.6
	7.0	0.051 ± 0.001	2.953 ± 0.013	1.273 ± 0.007	5.218 ± 0.020	3.608 ± 0.038	0.208 ± 0.004	4.06 ± 0.08	0.096 ± 0.001	104.1 ± 2.3	6.03 ± 0.04	28.9 ± 0.6
IMZ351	5.0	0.047 ± 0.001	3.485 ± 0.025	1.390 ± 0.005	6.620 ± 0.014	4.735 ± 0.039	0.190 ± 0.008	4.00 ± 0.17	0.081 ± 0.001	110.1 ± 4.7	7.38 ± 0.04	38.9 ± 1.6
	6.0	0.047 ± 0.001	3.074 ± 0.017	1.223 ± 0.006	5.825 ± 0.028	4.404 ± 0.046	0.183 ± 0.003	3.91 ± 0.06	0.085 ± 0.001	100.8 ± 1.7	6.73 ± 0.05	36.7 ± 0.6
	7.0	0.048 ± 0.001	2.826 ± 0.031	1.239 ± 0.004	5.081 ± 0.009	3.639 ± 0.053	0.187 ± 0.003	3.88 ± 0.06	0.095 ± 0.001	90.7 ± 1.8	5.99 ± 0.05	32.0 ± 0.5

#### Intracellular metabolite concentrations

IMZ351 showed decreased biomass N-content when compared to IME169, suggesting that deletion of Mep genes resulted in an altered cellular response in nitrogen-limited chemostat cultures. To investigate physiological effects caused by the decreased specific NH_X_ uptake rates, the concentrations of intracellular metabolites involved in carbon and nitrogen metabolism were measured (Additional file 1). While, surprisingly, the intracellular NH_X_ concentration was actually significantly higher in IMZ351, the intracellular concentration of the product of the most prominent entry route for NH_X_ assimilation, L-glutamate (Glu), was comparable in both strains at each pH. The L-glutamine concentration, which is the end product of the alternative route of NH_X_ assimilation via the GS-GOGAT system, was lower for IMZ351 compared to the reference strain, but increased with pH. Downstream, the concentration of amino acids synthesized in the mitochondria -L-alanine, L-valine and L-lysine- were significantly lower in IMZ351. Furthermore, the intracellular trehalose concentration -which is an indicator of cellular stress and/or nitrogen limitation [46]- was significantly higher in IMZ351 at all pH conditions.

#### Effects of NH_3_ diffusion on the protein levels

Alteration of the NH_X_ transport mechanism resulted in changes in cellular metabolism, which were also related to changes in the protein levels [47, 48]. The measurement of relative protein levels showed changes in more than 300 different proteins, but in amounts that varied between strains in the different pH conditions. The concentration of certain proteins were low and could only be observed in one of the strains. Those proteins are called from “unique proteins”, although in this case the word “unique” does not imply that they are totally absent from the other strains/conditions, but only that their levels are in some cases below the detection tershold. While our analytical method cannot provide an answer on whether proteins are present or absent in the protein levels, then, these ‘unique’ proteins can be considered a especial subset of up/down regulated proteins. Nineteen proteins were consistently found as unique in IMZ351, but not in the reference strain (IME169) at all pH conditions; i.e., they were expressed at measurable levels in IMZ351 while not in IME169 (Additional file 1). Of these, of particular interest were Rav1 (regulator of the activity of vacuolar ATPase acitivty), Hog1 (global regulator of stress responses), and Mck1 (threonine/serine protein kinase that regulates DNA replication [49], C-metabolism, and protein kinase A activity [50]).

GO-term cluster analysis revealed that among the proteins with at least 50% increased levels in IMZ351 were related to stress-response terms, i.e., DNA replication stress and inefficient DNA replication [51], as well as autophagy and decreased protein production [52]. In that group, Rtp6 and Cps1 were found at higher levels and described to correlate with severe N-limitation state. While a significant up-regulation of proteins involved in various stress responses was observed, no significant differences in proteins involved in nitrogen catabolite repression (NCR) and central nitrogen metabolism were observed.

## Discussion

Based on the experimental and modeling results, it was shown that NH_3_ diffusion is the main NH_X_ transport mechanism in Mep-deficient strain IMZ351. Alternative mechanisms, like transport through K^+^-channels, can be excluded. In particular, the aerobic micro-titer experiments showed that the growth rate was dependent on extracellular NH_3_ concentration rather than the electrochemical gradient. Furthermore, the cytosolic/extracellular ratio of NH_X_ for IMZ351 under aerobic N-limiting conditions was consistent with the ratio predicted for NH_3_ diffusion, but not with any transport mechanism dependent on the cell membrane potential or pmf.

By contrast with IMZ351, the observed IC/EC ratio for IME169 remained relatively constant and at least one order of magnitude higher than the ratios observed in IMZ351 across all pH values. Nevertheless, the experimental ratios did not match the predicted ratios at pH 6 and pH 7, which could be explained by a limitation in affinity (*K*_*M*_) of the Mep proteins rather than the thermodynamic driving force. Notwithstanding this, our results for IME169 at different pH values clearly show that NH_4_^+^ is the transported species, opposing previous studies suggesting that Mep proteins and other Amt-class transporters carry uncharged NH_3_ across the membrane [42, 53].

The metabolic profile in both strains presented clear differences, like a significantly higher concentration of intracellular NH_X_ and trehalose in the strain IMZ351. While the cause of this remains unanswered, the observation raises questions about the signaling pathways for N-limitation. Our experimental results suggest that intracellular NH_X_ is not involved in signaling.

**Fig. 2:**
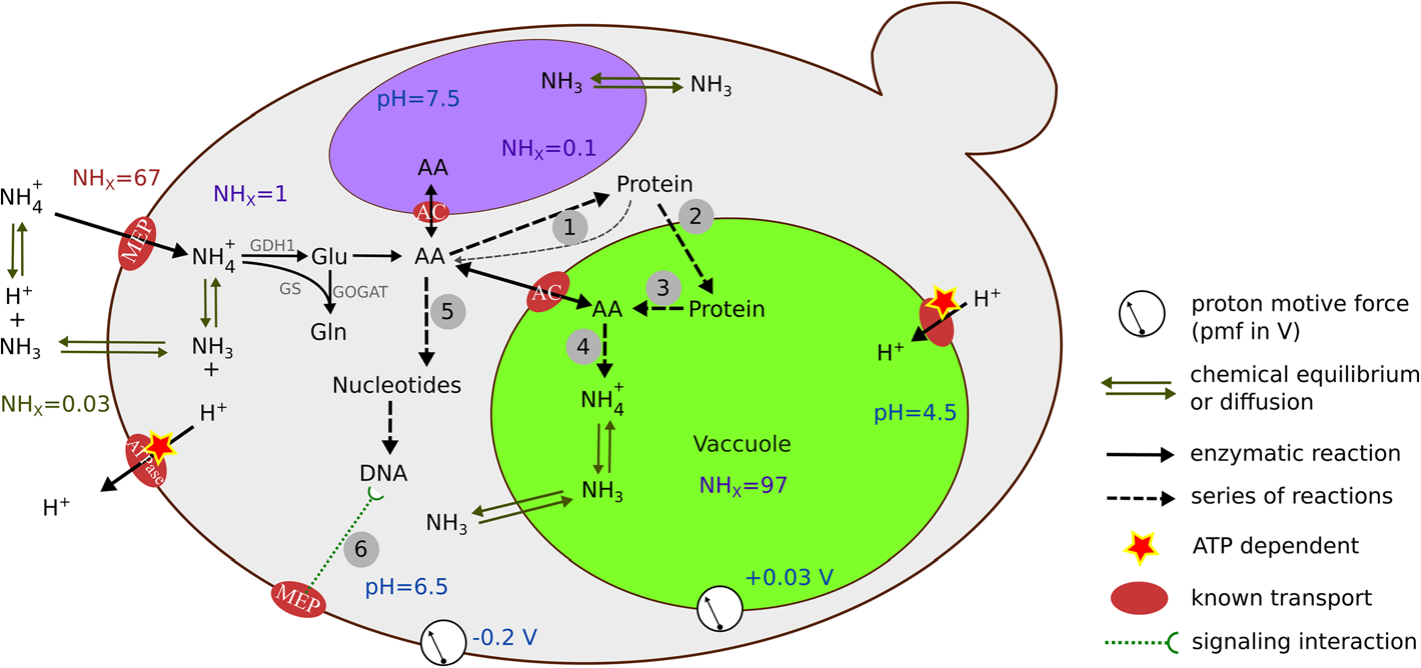
Summary of mechanisms and hypotheses for IME169 (reference strain) and IMZ351 (Δmep). For both strains, the main reactions of nitrogen incooperation are GS-GOGAT, Gdh1, Gdh2 and Gdh3, fueling amino acids and biomass synthesis, esp. protein (1) and nucleotides (5). The NH4 transporter proteins Mep1 and Mep2 work as transceptors (6), signaling the presence of ammonium and activate a yet unidentified signaling cascade [57], possibly protein kinase A (PKA) as described in previous works [54, 57]. In the absence of MEP-proteins there is a constitutive down-regulation/repression of PKA and up-regulation/activation of genes associated with autophagy and DNA replication stress (see main text). These stress responses lead to decreased DNA replication and protein biosynthesis and increased protein turnover (3). The protein turnover could result in production of amino acids and/or higher NHX concentrations (4), amino acid recycling and trehalose overproduction

Proteomic analysis revealed significantly higher levels of proteins related to recycling of N-compounds (proteins, amino acids) and general cellular stress responses, suggesting an altered cellular response to N-limitation. However, in view of the higher intracellular NH_X_ concentration (Table 2) and the generally comparable concentrations of most intracellular N-based metabolites (Additional file 1), this appears to be unrelated to any particular signaling metabolite in the intracellular space. Mep1 and Mep2 have been described as NH_4_^+^ transceptors, not only responsible for transport across the cell membrane but also acting as cAMP-independent activators of the protein kinase A (PKA) signaling cascade; this signal is triggered due to conformational changes in Mep1 and Mep2 after binding with ammonium [54]. In the absence of extracellular NH_4_^+^, no ammonium permease-mediated signal is sent to the PKA complex, leading to its inactivation and subsequent repression of glycolytic genes and of genes involved in cellular growth and proliferation, and in particular to an up-regulation of genes responsible for the cellular stress response mediated by STRE (stress response element) [55]. This hypothesis is supported indirectly by the presence of Mck1, which was one of the proteins only found in IMZ351 but not in the reference strain.Mck1 is a known transcriptional regulator, PKA inhibitor, and modulator of other cellular processes, such as DNA replication and protein degradation. We thus speculate that a constitutive up-regulation of the cellular stress response is generated upon deletion of the genes encoding the ammonium permeases. Proteins involved in various stress responses, in particular DNA replication stress, decreasing protein synthesis, increasing protein turnover, and increased cell-wall protective agents (trehalose, cell wall repair systems) [56] are expressed in IMZ351 (Fig. 2). However, whether this fully explains the metabolite profile of IMZ351 and especially the increase in intracellular NH_X_ and the decrease in mitochondrial amino acids, or whether additional responses are also involved is yet to be ascertained.

This (stressed) phenotype of IMZ351 revealed the system’s nature –while the cost for the transport could be reduced, secondary responses lead to ATP consumption and the aim of improved energy efficiency cannot be achieved without additional steps. This increased energy consumption interferes with the ability to apply anaerobic production conditions without decreasing the negative physiological effects from deletion of Mep proteins.

## Conclusions

The underlying goal of this study was to engineer membrane potential-decoupled NH_X_ assimilation for use in bulk N-containing chemical production. Although diffusion of NH_3_ metabolically conserves ATP in the N-assimilation process, the observed metabolic rates did not show this energy conservation improvement. The different degrees of N-limitation in both strains led to an uncoupling between of metabolic ATP saving from biomass production, as observed from the experimental N-biomass content, trehalose concentration and *q*_*ATP*_ */q*_*N*_ ratio.

To enable future industrial (anaerobic) applications, elucidation and subsequent engineering of this stress response will be required.

## Additional file

Additional file 1: Contains details on strain construction and confirmation, additional details on calculations and additional metabolome and proteome measurements. (PDF 1095 kb)

## Abbreviations

NH_3_: Ammonia
NH_4_^+^: Ammonium
NH_X_: Sum of NH_3_ + NH_4_^+^ (total ammonium)
P_1a_: Permeability coefficient
PKA: Protein kinase A
pmf: Proton motive force
STRE: Stress response element
UHPLC-IDMS: Ultra-high performance liquid chromatography with isotope dilution mass spectrometry
