# Supplementary material for: Membrane potential independent transport of NH3 in the absence of ammonium permeases in Saccharomyces cerevisiae
Source: BMC Syst Biol. 2017 Apr 17;11:49. doi: 10.1186/s12918-016-0381-1 (PMC5392931; doi:10.1186/s12918-016-0381-1)
Supplement: Additional file 1: — Contains details on strain construction and confirmation, additional details on calculations and additional metabolome and proteome measurements. (PDF 1095 kb) [file 12918_2016_381_MOESM1_ESM.pdf]

Membrane potential independent transport of NH_3 in the absence of ammonium permeases in *Saccharomyces cerevisiae*

Additional File 1

1. Construction and confirmation of *mep1,2,3*Δ in *S. cerevisiae*

To investigate the role of *MEP1*, *MEP2* and *MEP3* in ammonium assimilation and the effects of deletion on cell physiology, *MEP1*, *MEP2* and *MEP3* were sequentially deleted using the Cre/loxP recombinase system from CEN.PK113-3B. The genotype of the resulting prototrophic strain IMZ351 (*ura3-52, his3-D1, mep1*Δ, *mep2*Δ, *mep3*Δ pUDE199) and the corresponding prototrophic *MEP1*, *MEP2*, *MEP3* positive control strain IME169 (*ura3-52, his3-D1*, *MEP1, MEP2, MEP3* pUDE199) were confirmed by diagnostic PCR (figure S1) then used for further analysis.

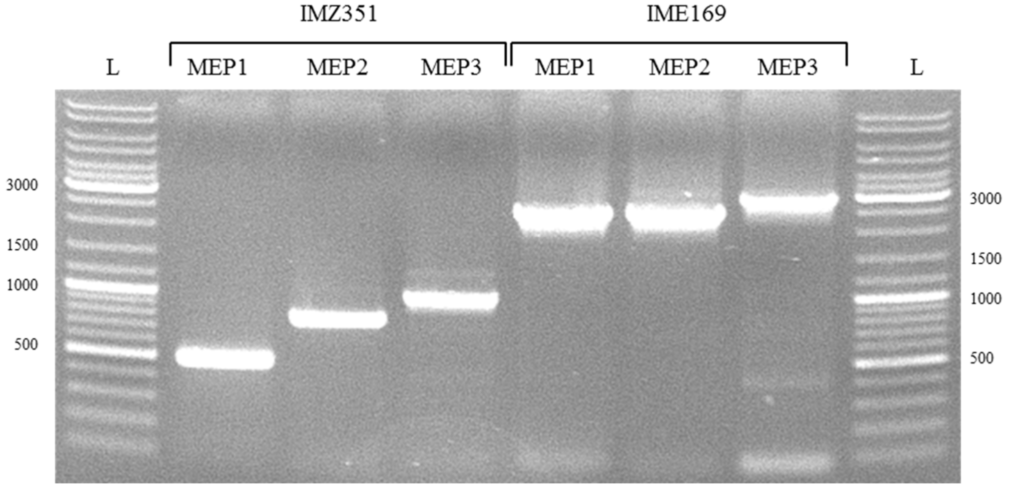

Figure S1. PCR analysis of the MEP1, MEP2 and MEP3 regions of IMZ351 and IME169. PCR bands were generated using primers which bound in the upstream and downstream region of each gene with different sized bands between IMZ351 and IME169 confirming the deletion of each gene. L: DNA ladder.

2. Theoretical calculations.

2.1. Cytosol/Extracellular ammonium equilibrium ratios

Thermodynamic equilibrium of NH_4^+ uniport transport (no compartmentalization)
If ammonium is transported as single positively charged species using an electrochemical gradient as driving force, and it is assumed that it is evenly distributed in the intracellular space, the in/out equilibrium ratio of ammonium is described by equation S1.

$$\frac{[NH_X]_{IC}}{[NH_X]_{EC}} = \left(\frac{1+10^{pH_{cyt}-pKa}}{1+10^{pH_{EC}-pKa}} \right) \times \exp\left(-\frac{F \times \Delta\psi_m}{R \times T} \right) \qquad (S1)$$

With,

$$\Delta\psi_m = -pmf + 2.303 \times \frac{R \times T}{F} \times \left(pH_{in} - pH_{out} \right) \qquad (S2)$$

Where F is the Faraday's constant, pmf is the proton motive force (assumed $-pmf$ = -200 mV), R the universal gas constant, T the absolute temperature in K, pH_{cyt} and pH_{EC} are the cytosolic and extracellular pH, respectively; the pKa for ammonium is 9.25. When pH_{cyt} and pH_{EC} are assumed to be 6.5 [1] and 5, respectively; the in/out ratio is 67 at an absolute temperature of 303.15K; this indicates that at equilibrium most of the ammonium will be found in the intracellular space.

2.1.1. Equilibrium of NH_3 diffusion (no compartmentalization)

On the other hand, if diffusion is the main transport mechanism the in/out equilibrium ratio of NH_X is dependent on the concentration of ammonia (NH_3) in both sides of the membrane, as it is the only species that can cross the cytosolic membrane without transporter. The in/out equilibrium of ammonia is dependent on the cytosolic (pH_{cyt}) and the extracellular pH (pH_{EC}), if there is no compartmentalization of ammonium, equation S3 describes the equilibrium between $[NH_X]_{IC}$ and $[NH_X]_{EC}$.

$$\frac{[NH_X]_{IC}}{[NH_X]_{EC}} = \left(\frac{1+10^{pKa-pH_{cyt}}}{1+10^{pKa-pH_{EC}}} \right) \qquad (S3)$$

Assuming similar conditions as in the case of *1.1.1 Equilbrium of NH_4^+ uniport transport*, the in/out ratio is 0.03, in this case at equilibrium most of the ammonium will be present in the extracellular space.

2.1.2. Effect of compartmentalization

In sections 1.1.1 and 1.1.2 it was considered that ammonium would be evenly distributed in the cell. However, previous studies [2] suggest large accumulation of NH_X in the intracellular space that do not correspond with a NH_4^+-uniport transport mechanism.
It was shown [2] that *S. cerevisiae* cells defective on V-ATPase activity accumulate significantly lower concentrations of ammonium compared to strains with functional V-ATPase; which is good

evidence to hypothesize that NH_X is transported into the vacuole as NH_3 and due to the acid environment in that compartment is protonated again into NH_4^+ creating significant accumulation of NH_X in the vacuole (figure S2); due to the basic pH in the mitochondrion, it is unlikely that NH_X is accumulated in large quantities in that compartment. Compartmentalization of ammonium is critical, as it will change the expected intracellular/extracellular ratios significantly.
Vacuoles could occupy as much as 25% of the cell volume [3]. The most recent measurements suggest that an average of 14% can be assumed [4] and the cytosolic volume corresponds to 70% of the cell volume [4]. Additionally, V-ATPase can sustain a pH difference between cytosol and vacuole of 1-2 pH units, typically pH_{cyt}=6.5 and pH_{vac}=4.5; therefore, vacuolar compartmentalization could significantly affect the in/out equilibrium ratio of NH_3-diffusion (figures S3).

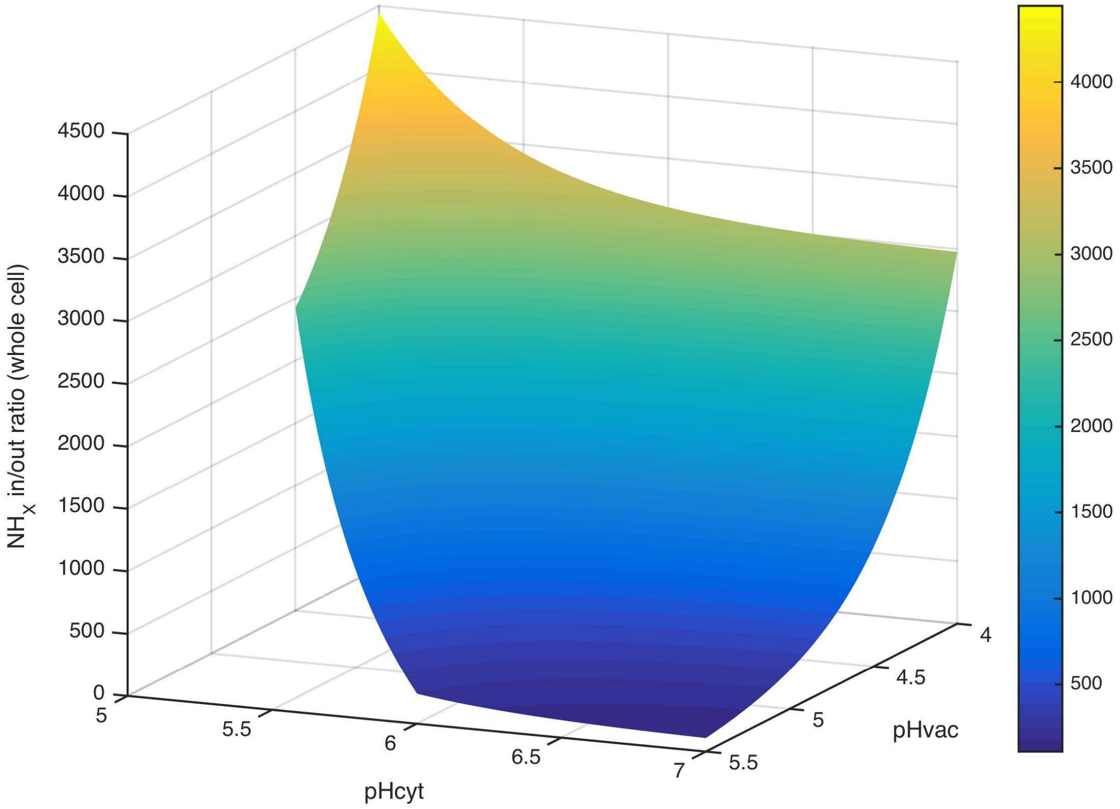

Figure S2. In/out NH_X equilibrium ratios at pH_{out}=5. It is considered that NH_4^+-uniport is the transport mechanism for NH_X, and compartmentalization of the molecule into the vacuole. Only in/out ratios for the case pH_{cyt}> (pH_{vac}+0.5) are plotted. Color code is based on the NH_X in/out ratios (z-axis).

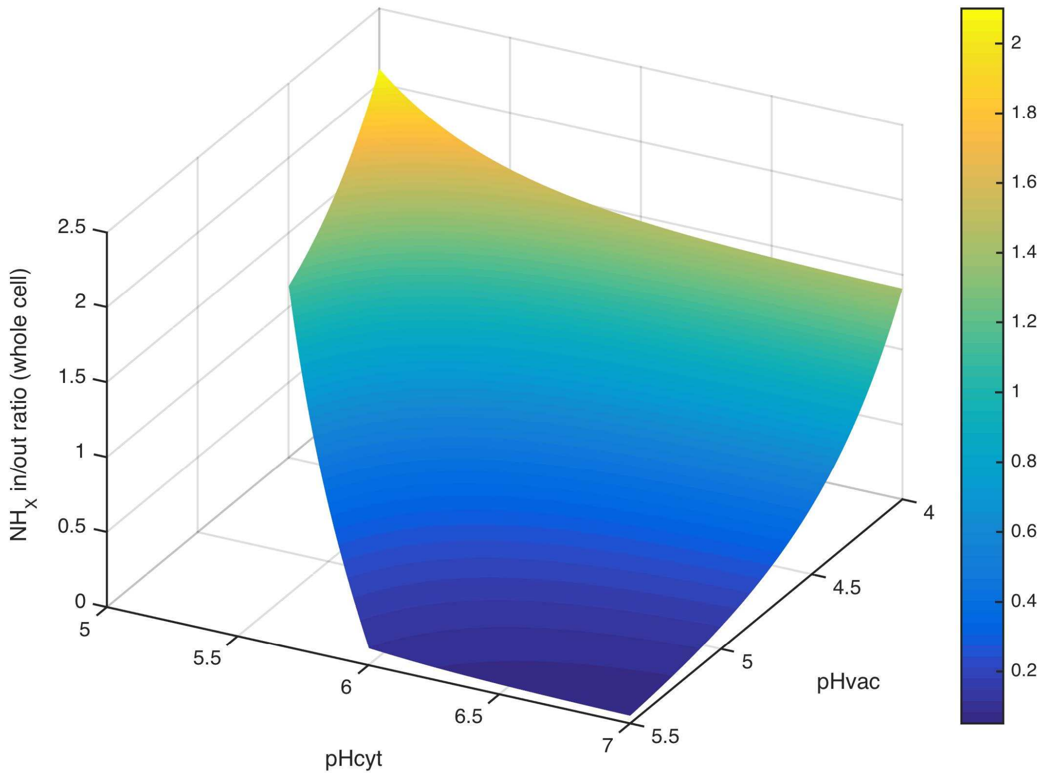

Figure S3. In/out NH_X equilibrium ratios at pH_{out}=5. It is considered that NH_3-diffusion is the transport mechanism for NH_X, compartmentalization into the vacuole is assumed. In/out ratios for the condition pH_{cyt}> (pH_{vac}+0.5) are shown. Color code is based on the NH_X in/out ratios (z-axis).

2.2. Theoretical calculation of permeability coefficient of ammonium in *Saccharomyces cerevisiae*.

Ammonia (NH_3) is transported across cell membranes using passive diffusion [5], different works measured the apparent permeability coefficient of ammonia in synthetic bilayer lipid membranes (48×10^{-3} cm/s) [6] and erythrocyte membranes (53×10^{-3} cm/s) [7]. The theoretical permeability coefficient could be calculated with the $-q_N = P_{1a} \times a_m \times([NH_3]_{EC}-[NH_3]_{cyt})$ and the maximum growth rate in *S. cerevisiae*, when NH_4^+ is used as N-source and glucose as C- and energy source at pH_{EC}=5, μ^{max} is around 0.35 h^{-1}. The N content in biomass is 0.148 mol/CmolX [8], which is equivalent to 5.60 $\times10^{-3}$ mol N/g_{CDW}; this leads to a $-q_{NH4+}^{max}$ = 1.96 $\times10^3$ µmol N/g_{CDW}. Assuming that $[NH_3]_{cyt}$ << $[NH_3]_{EC}$, a_m=3.22 m^2/g_{CDW} and a typical NH_X concentration of 75 mmol/L ($[NH_3]_{EC}$ = 4.21 µmol/L); the estimated apparent permeability coefficient of NH_3 for *S. cerevisiae* will be 0.144 m/h (4.02 $\times10^{-3}$ cm/s).

Although, many reactions would require a minimal amount of ammonium, therefore $[NH_X]_{cyt}$ will never be zero. The assumption of negligible $[NH_3]_{cyt}$ is realistic, based on the equilibrium of the reaction glutamate dehydrogenase NADPH-dependent (GDH1) at cytosolic pH (pH_{cyt}=6.5), typical intracellular glutamate concentration of 35 mmol/L_{IC}, AKG concentration of 0.6 mmol/L_{IC}, and

NADPH/NADP ratio of 22 [9]. We estimated a minimal NH_X concentration in the intracellular space of 0.33 µmol/L_{IC} (5.0 ×10^{-4} µmol/L_{IC}), which is much lower than the extracellular NH_3. This calculation shows that even an apparent permeability coefficient for NH_3 10 times lower than the reported for other systems should be sufficient to sustain fast growth rates in *Saccharomyces cerevisiae*, if NH_3-diffusion is the nitrogen uptake mechanism.

2.3. Estimation of cytosolic NH_X concentration and P_{1a} using experimental results.

As explained in the main text, estimation of the cytosolic NH_X concentration requires the whole cell NH_X content, compartment pH and volumes. The first equation used to obtain the intracellular NH_X distribution is the total intracellular NH_X balance (equation S4), in S4 the ratios V_{cyt}/V_{cell}, V_{vac}/V_{cell}, and V_{mit}/V_{cell} express the volume fraction that each compartment represents with respect to the total cell volume; the values used in the text are 0.7, 0.14 and 0.01, respectively.

$$\frac{V_{cyt}}{V_{cell}}\times\left[NH_X\right]_{cyt}+\frac{V_{vac}}{V_{cell}}\times\left[NH_X\right]_{vac}+\frac{V_{mit}}{V_{cell}}\times\left[NH_X\right]_{mit}-\left[NH_X\right]_{IC}=0 \quad \text{(S4)}$$

Additionally, it is necessary to assume that NH_3 diffusion is the transport mechanism between compartments, and this process is so fast that can be considered at thermodynamic equilibrium; equation S5 expresses the equilibrium for the transport between cytosol and vacuole, and equation S6 expresses the equilibrium for the transport process between cytosol and mitochondria.

$$\frac{1+10^{pKa-pH_{vac}}}{1+10^{pKa-pH_{cyt}}}\times\left[NH_X\right]_{cyt}-\left[NH_X\right]_{vac}=0 \quad \text{(S5)}$$

$$\frac{1+10^{pKa-pH_{mit}}}{1+10^{pKa-pH_{cyt}}}\times\left[NH_X\right]_{cyt}-\left[NH_X\right]_{mit}=0 \quad \text{(S6)}$$

Finally, the kinetic expression of $-q_N$, which is the mathematical expression for NH_3 diffusion from the extracellular space to the cytosol (S7), completes a system of 4 algebraic equations.

$$-q_N+P_{1a}\times a_m\times\left(\left[NH_X\right]_{EC}-\left[NH_X\right]_{cyt}\right)=0 \quad \text{(S7)}$$

This system of equations can be solved numerically using the experimentally measured variables $[NH_X]_{EC}$, $[NH_X]_{IC}$, and $-q_N$; assumptions about compartment volumes and pH (pH_{cyt}, pH_{vac} and pH_{mit}); being the four variables to determine $[NH_X]_{cyt}$, $[NH_X]_{mit}$, $[NH_X]_{vac}$ and P_{1a}.

3. Metabolomic analysis of IMZ351 and IME169

Table S1. Metabolite concentrations measured in strains IMZ351 and IME169 under aerobic N-limited chemostat conditions (D=0.05 h^{-1}).

Metabolite		Whole cell concentration (mmol/L_{IC})					
		IMZ351			IME169		
		pH = 5	pH = 6	pH = 7	pH = 5	pH = 6	pH = 7
Glutamate	CHEBI: 29985	33.075 ± 1.337	40.889 ± 0.750	41.098 ± 1.513	35.667 ± 0.520	39.681 ± 0.576	45.336 ± 0.350
Glutamine	CHEBI: 18050	8.776 ± 0.355	8.812 ± 0.162	7.877 ± 0.290	11.253 ± 0.164	9.333 ± 0.136	6.090 ± 0.047
Aspartate	CHEBI: 29991	5.363 ± 0.217	6.001 ± 0.110	6.454 ± 0.238	5.922 ± 0.086	5.757 ± 0.084	6.738 ± 0.052
Lysine	CHEBI: 18019	5.041 ± 0.204	5.172 ± 0.095	2.959 ± 0.109	7.063 ± 0.103	6.806 ± 0.099	5.530 ± 0.043
Alanine	CHEBI: 16977	11.810 ± 0.477	11.387 ± 0.209	8.073 ± 0.297	16.963 ± 0.247	14.584 ± 0.212	11.558 ± 0.089
Valine	CHEBI: 16414	2.320 ± 0.094	2.552 ± 0.047	1.857 ± 0.068	2.764 ± 0.040	2.815 ± 0.041	2.492 ± 0.019
Arginine	CHEBI: 16467	8.403 ± 0.340	12.702 ± 0.226	6.706 ± 0.247	9.677 ± 0.141	12.479 ± 0.182	13.466 ± 0.094
AKG	CHEBI: 16810	0.388 ± 0.016	0.648 ± 0.012	0.831 ± 0.031	0.604 ± 0.009	0.864 ± 0.013	1.389 ± 0.011
Trehalose	CHEBI: 16551	147.79 ± 5.97	180.19 ± 3.30	175.17 ± 6.45	95.72 ± 1.40	115.64 ± 1.68	131.06 ± 1.01
NAD	CHEBI: 57540	1.220 ± 0.070	1.042 ± 0.026	0.709 ± 0.037	0.874 ± 0.018	0.760 ± 0.016	0.656 ± 0.006
NADH	CHEBI: 57945	0.013 ± 0.001	0.027 ± 0.001	0.017 ± 0.001	0.027 ± 0.001	0.041 ± 0.001	Not detected
NADP	CHEBI: 58349	0.299 ± 0.017	0.304 ± 0.008	0.197 ± 0.010	0.219 ± 0.004	0.225 ± 0.005	0.244 ± 0.002
NADPH	CHEBI: 57783	0.026 ± 0.002	0.021 ± 0.001	0.017 ± 0.001	0.025 ± 0.001	0.021 ± 0.001	0.017 ± 0.001
ADP	CHEBI: 29985	0.879 ± 0.036	0.936 ± 0.017	0.536 ± 0.020	0.704 ± 0.010	0.733 ± 0.011	0.677 ± 0.005
ATP	CHEBI: 30616	2.810 ± 0.114	2.711 ± 0.050	1.614 ± 0.059	2.188 ± 0.032	2.047 ± 0.030	1.908 ± 0.015
Energy charge		0.799 ± 0.041	0.790 ± 0.018	0.780 ± 0.036	0.793 ± 0.015	0.775 ± 0.014	0.795 ± 0.008

4. Proteomic analysis of IMZ351 and IME169

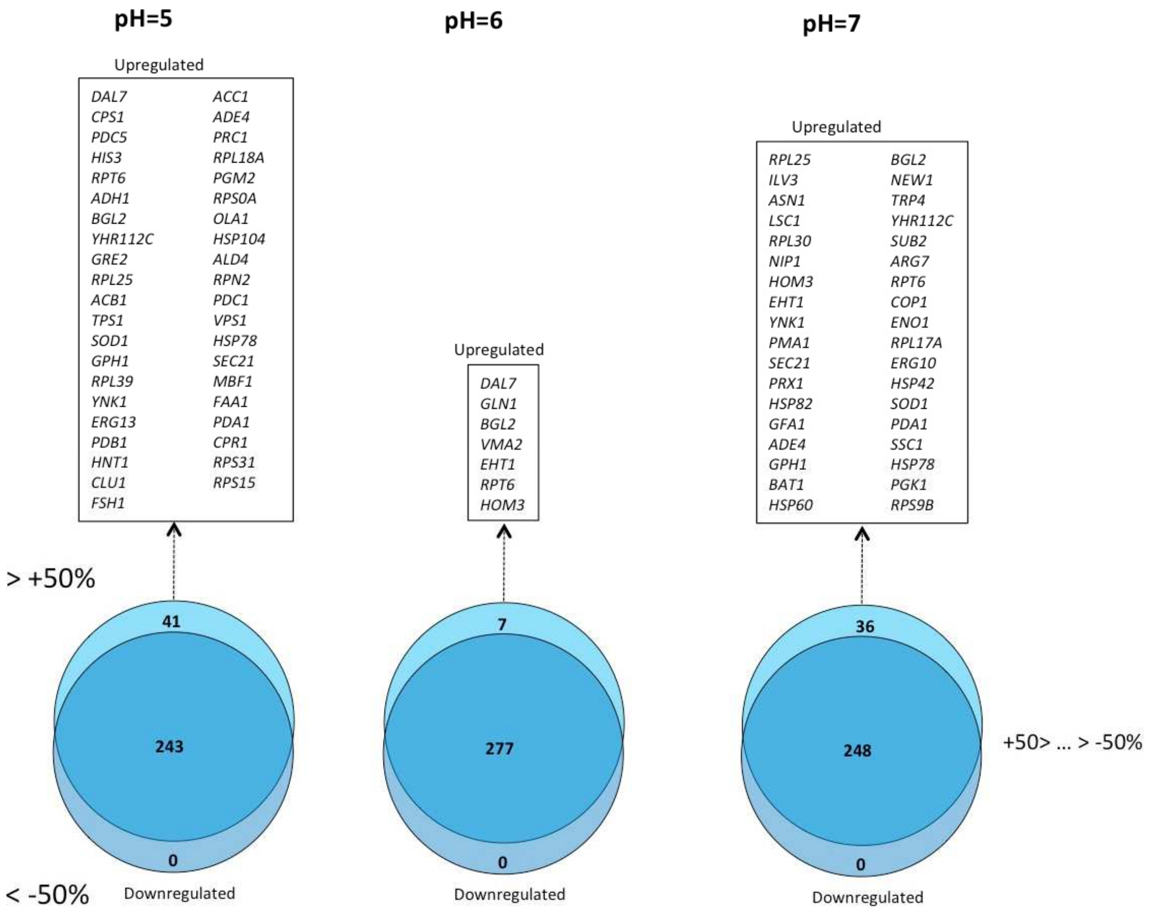

Figure S4. Changes in protein expression found in strain IMZ351 compared to IME169 under aerobic N-limited chemostat conditions (D=0.05 h^{-1}) at individual pH conditions. Protein expression is considered up regulated when the relative change in protein content is 50% or higher with respect to the reference strain (IME169), at the same environmental condition. Bgl2 and Rpt6 are consistently found up regulated in all cases.

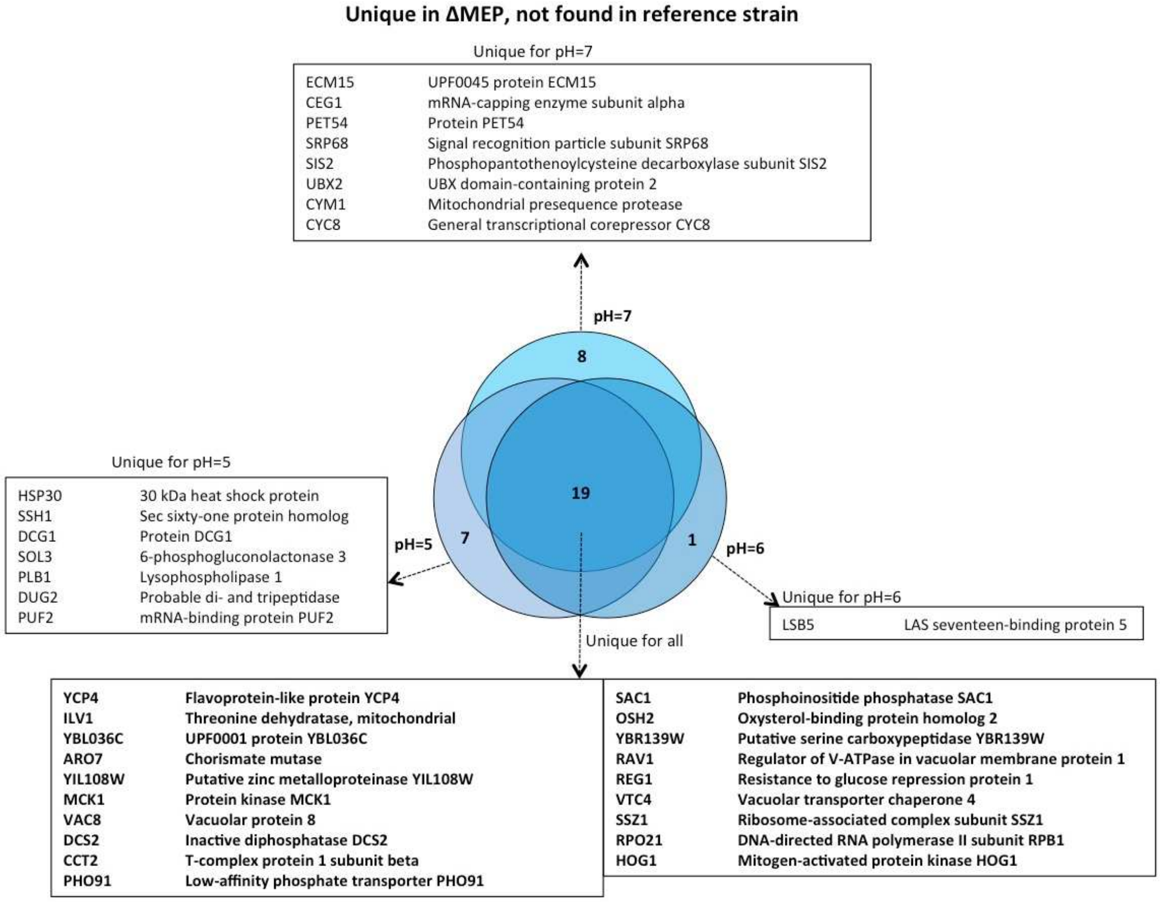

Figure S5. Proteins identified with two or more confidence peptides in the strain IMZ351, but not found in IME169 growing under aerobic N-limited chemostat conditions (D=0.05 h^{-1}) at every pH condition are considered "unique proteins". Unique proteins are in a sense a especial type of up regulated proteins, as their concentration was high enough to be found in IMZ351 extracts but not in IME169; this does not necessarily imply that they are absent in IME169.

References.

1 Kresnowati, M. T., Suarez-Mendez, C., Groothuizen, M. K., van Winden, W. A. & Heijnen, J. J. Measurement of fast dynamic intracellular pH in *Saccharomyces cerevisiae* using benzoic acid pulse. *Biotechnol. Bioeng.* **97**, 86-98, doi:10.1002/bit.21179 (2007).

2 Wood, C. C., Poree, F., Dreyer, I., Koehler, G. J. & Udvardi, M. K. Mechanisms of ammonium transport, accumulation, and retention in ooyctes and yeast cells expressing *Arabidopsis AtAMT1;1*. *FEBS Lett.* **580**, 3931-3936, doi:10.1016/j.febslet.2006.06.026 (2006).

3 Perktold, A., Zechmann, B., Daum, G. & Zellnig, G. Organelle association visualized by three-dimensional ultrastructural imaging of the yeast cell. *FEMS Yeast Res.* **7**, 629-638, doi:10.1111/j.1567-1364.2007.00226.x (2007).

4 Uchida, M. *et al.* Quantitative analysis of yeast internal architecture using soft X-ray tomography. *Yeast* **28**, 227-236, doi:10.1002/yea.1834 (2011).

5 Kleiner, D. The transport of NH_3 and HN_4^+ across biological membranes. *Biochimica et Biophysica Acta (BBA) - Reviews on Bioenergetics* **639**, 41-52, doi:10.1016/0304-4173(81)90004-5 (1981).
6 Antonenko, Y. N., Pohl, P. & Denisov, G. A. Permeation of ammonia across bilayer lipid membranes studied by ammonium ion selective microelectrodes. *Biophys. J.* **72**, 2187-2195, doi:10.1016/S0006-3495(97)78862-3 (1997).
7 Labotka, R. J., Lundberg, P. & Kuchel, P. W. Ammonia permeability of erythrocyte membrane studied by ^{14}N and ^{15}N saturation transfer NMR spectroscopy. *Am J Physiol* **268**, C686-699 (1995).
8 Lange, H. C. & Heijnen, J. J. Statistical reconciliation of the elemental and molecular biomass composition of *Saccharomyces cerevisiae*. *Biotechnol. Bioeng.* **75**, 334-344, doi:10.1002/bit.10054 (2001).
9 Zhang, J. *et al.* Determination of the Cytosolic NADPH/NADP Ratio in *Saccharomyces cerevisiae* using Shikimate Dehydrogenase as Sensor Reaction. *Sci Rep* **5**, 12846, doi:10.1038/srep12846 (2015).
